# Clinical efficacy and safety of multipotent adult progenitor cells (invimestrocel) for acute respiratory distress syndrome (ARDS) caused by pneumonia: a randomized, open-label, standard therapy–controlled, phase 2 multicenter study (ONE-BRIDGE)

**DOI:** 10.1186/s13287-023-03451-z

**Published:** 2023-08-22

**Authors:** Kazuya Ichikado, Toru Kotani, Yasuhiro Kondoh, Hideaki Imanaka, Takeshi Johkoh, Kiminori Fujimoto, Shin Nunomiya, Tomotaka Kawayama, Masanori Sawada, Eric Jenkins, Sadatomo Tasaka, Satoru Hashimoto

**Affiliations:** 1 Division of Respiratory Medicine, Social Welfare Organization Saiseikai Imperial Gift Foundation, Inc., Saiseikai Kumamoto Hospital, 5-3-1 Chikami, Minami-ku, Kumamoto City, 8614101 Japan; 2 Department of Intensive Care Medicine, Showa University School of Medicine, Tokyo, Japan; 3 Department of Respiratory Medicine and Allergy, Tosei General Hospital, Seto, Aichi Japan; 4 Department of Emergency Medicine, Takarazuka City Hospital, Takarazuka, Hyogo Japan; 5 Department of Radiology, Kansai Rosai Hospital, Amagasaki, Hyogo Japan; 6 Department of Radiology, Kurume University School of Medicine, Fukuoka, Japan; 7 Division of Intensive Care, Department of Anesthesiology and Intensive Care Medicine, Jichi Medical University School of Medicine, Tochigi, Japan; 8 Division of Respirology, Neurology, and Rheumatology, Department of Medicine, Kurume University School of Medicine, Fukuoka, Japan; 9 Healios K.K., Tokyo, Japan; 10 Athersys, Inc., Cleveland, OH USA; 11 Department of Respiratory Medicine, Hirosaki University Graduate School of Medicine, Aomori, Japan; 12 Department of Anesthesiology and Intensive Care Medicine, Kyoto Prefectural University of Medicine, Kyoto, Japan; 13 Present Address: Department of Intensive Care, Yokosuka General Hospital Uwamachi, Kanagawa, Japan; 14 Present Address: Kiniksa Pharmaceuticals, Lexington, MA USA

**Keywords:** Acute respiratory distress syndrome, Lung injury, Mesenchymal stem cells, Pneumonia

## Abstract

**Background:**

Acute respiratory distress syndrome (ARDS) is a life-threatening inflammatory lung injury with high mortality; no approved medication exists. Efficacy and safety of bone marrow–derived, allogeneic, multipotent adult progenitor cells (invimestrocel) plus standard treatment in patients with ARDS caused by pneumonia was evaluated.

**Methods:**

A randomized, open-label, standard therapy–controlled, phase 2 study (January 2019–September 2021) conducted in 29 centers in Japan. Patients with ARDS caused by pneumonia, with extensive early fibroproliferation on high-resolution computed tomography and low risk of systemic organ failure identified by an Acute Physiology and Chronic Health Evaluation (APACHE II) score were included. Patients were randomized 2:1 to receive a single intravenous infusion of 9.0 × 10^8^ cells of invimestrocel (administered at a rate of up to 10 mL/min over 30–60 min by free flow) plus standard treatment (N = 20) or standard treatment (N = 10) consistent with the clinical practice guidelines of the Japanese Respiratory Society for the management of ARDS. Primary endpoint was ventilator-free days (VFDs) through day 28 after study treatment. Analysis of covariance was performed with treatment group, age, partial pressure arterial oxygen/fraction of inspired oxygen ratio, and APACHE II score as covariates.

**Results:**

Median (interquartile range) number of VFDs was numerically higher in the invimestrocel group versus standard group (20.0 [0.0–24.0] vs 11.0 [0.0–14.0]) but was not statistically significantly different (least square [LS] means [95% confidence interval (CI)]: invimestrocel group, 11.6 [6.9–16.3]; standard group, 6.2 [− 0.4 to 12.8]; LS mean difference [95% CI], 5.4 [− 1.9 to 12.8]; *p* = 0.1397). Ventilator weaning rate at day 28 was 65% (13/20) versus 30% (3/10), and mortality rate was 21% (4/19) versus 29% (2/7) at day 28 and 26% (5/19 patients) versus 43% (3/7 patients) at day 180, for the invimestrocel and standard groups, respectively. No allergic or serious adverse reactions were associated with invimestrocel.

**Conclusions:**

In Japanese patients with ARDS caused by pneumonia, invimestrocel plus standard treatment resulted in no significant difference in the number of VFDs but may result in improved survival compared with standard treatment. Invimestrocel was well tolerated.

*Trial registration*: ClinicalTrials.gov, Identifier: NCT03807804; January 8, 2019; https://clinicaltrials.gov/ct2/show/NCT03807804.

**Supplementary Information:**

The online version contains supplementary material available at 10.1186/s13287-023-03451-z.

## Background

Acute respiratory distress syndrome (ARDS) is a life-threatening inflammatory lung injury that allows fluid to leak into the lungs and is often associated with multiple organ failure [1]. Lung injuries that can result in ARDS include sepsis, inhaling harmful substances, major trauma, and near drowning [1]. Pneumonia, including that resulting from COVID-19 caused by the severe acute respiratory syndrome coronavirus 2, can lead to ARDS [2]. Mortality due to ARDS remains high (~ 40%) [3], and survivors often have physical impairment and reduced quality of life (QoL) [4]. Pulmonary fibroproliferation caused by ARDS is one factor leading to mortality and functional impairment [5, 6]. No approved medication for ARDS exists, and therefore effective drug treatment options that reduce mortality and improve patients’ long-term QoL are required.

Preclinical studies using animal models of ARDS have demonstrated that treatment with mesenchymal stem cells (MSCs) can reduce the severity of acute lung injury [7–10] and that the MSCs act by modulating the inflammatory response associated with the acute lung injury [7–11]. Furthermore, several clinical trials investigating MSC treatment for ARDS showed that the MSC treatments were safe but larger trials are required for evaluation of efficacy [6, 12–15]. Similarly, multipotent adult progenitor cells [16] harvested from bone marrow were examined in a sheep model of ARDS and demonstrated modulation of the inflammatory response [9, 17]. In clinical studies in patients with myocardial infarction, stroke, and graft-versus-host disease, multipotent adult progenitor cells were well tolerated [18–20].

The stem cell product invimestrocel (MultiStem^®^) is composed of multipotent adult progenitor cells harvested from bone marrow of healthy unrelated consenting donors, which are then expanded individually in ex vivo culture to clinical scale [21]. Invimestrocel is considered to reduce inflammation, regulate immune system function, protect damaged or injured cells and tissues, promote angiogenesis, and promote tissue repair and healing [22]. The recently completed phase 1/2 MUST-ARDS study evaluated the safety of multipotent adult progenitor cells (invimestrocel) in patients with moderate-to-severe ARDS in the USA and the UK [23]. In the MUST-ARDS randomized, double-blind, placebo-controlled study, multipotent adult progenitor cells (invimestrocel) at a dose of 9.0 × 10^8^ cells were safe and well tolerated; 28-day mortality was lower, and median 28-day free from intensive care unit and ventilator-free days (VFDs) were higher, in the multipotent adult progenitor cell (invimestrocel) group versus the placebo group.

Clinical trials for treatments targeting biological pathways thought to be dysregulated in ARDS have been mostly inconclusive, and this may in part be due to the heterogeneity of ARDS [24]. In the MUST-ARDS study, the cause of ARDS included pneumonia, sepsis, and aspiration, and the severity of ARDS was moderate to severe (partial pressure arterial oxygen/fraction of inspired oxygen [PaO_2_/F_I_O_2_] < 200 mmHg [27 kPa]), consistent with the Berlin Definition [25]. The ONE-BRIDGE phase 2 study focused on ARDS caused by pneumonia only. The aim of the study was to further investigate the efficacy and confirm the safety of invimestrocel in Japanese patients and to explore changes in biomarkers and patient QoL. In ONE-BRIDGE, the severity of ARDS was defined by independent prognostic factors: the Acute Physiology and Chronic Health Evaluation (APACHE) II score and the severity of fibroproliferative lesions in the lung according to the high-resolution computed tomography (HRCT) score [5, 26].

## Methods

### Study design

This randomized, open-label, standard therapy–controlled, phase 2 multicenter study (ONE-BRIDGE) evaluated the efficacy of invimestrocel (multipotent adult progenitor cells/MultiStem^®^) plus standard treatment in patients with ARDS caused by pneumonia. The protocol was amended (April 9, 2020) to add a single-arm, open-label pilot study of invimestrocel plus standard treatment in patients with ARDS caused by COVID-19–induced pneumonia; however, the results of this pilot study are not included in this report. The study protocol and addendum were approved by the ethics review board of each site (Additional file 1: Table S1), and the study was conducted from January 1, 2019 to September 30, 2021. Written informed consent was provided by the patient or his/her legal representative. The study was conducted in accordance with the Declaration of Helsinki and in compliance with the Act on Securing Quality, Efficacy and Safety of Pharmaceuticals, Medical Devices, Regenerative and Cellular Therapy Products, Gene Therapy Products, and Cosmetics, Good Clinical Practice for Regenerative Medicine Products, and related laws and regulations. The study was registered at ClinicalTrials.gov (Identifier: NCT03807804).

### Study population

Complete inclusion and exclusion criteria are provided in Additional file 2. Patients were included if they were aged 20‒90 years, diagnosed with ARDS (according to the Berlin Definition [25]) caused by pneumonia, were receiving mechanical ventilation, and could receive the investigational product within 72 h after ARDS diagnosis. Patients who were at a high risk of progressive pulmonary fibroproliferation associated with secondary septic syndrome and could not be rescued by conventional treatments were included based on an estimated HRCT score ≥ 211 [5, 26]. Patients who were likely to die of severe systemic organ failure in a few days without confirming the effect of invimestrocel were excluded based on an APACHE II score ≥ 27 [27]. Patients were excluded if they had a life expectancy of < 6 months, were on mechanical ventilation for ≥ 1 week, or had suspected acute exacerbation of chronic pulmonary fibrosis, diffuse alveolar hemorrhage, chronic respiratory disease requiring continuous home oxygen therapy, severe chronic obstructive pulmonary disease (GOLD stage III [28] or higher), chronic pulmonary hypertension, a history of lobectomy, single-lung pneumonectomy, or lung transplantation, or severe chronic liver disease. Patients with ARDS due to trauma or other non-infectious factors such as pancreatitis were not included in this study.

### Randomization and treatment protocol

Patients were randomized 2:1 to receive either the investigational product invimestrocel plus standard treatment (invimestrocel group) or standard treatment (standard group) consistent with the clinical practice guidelines of the Japanese Respiratory Society for the management of ARDS [29] (Additional file 2). An interactive web response system was used for randomization; stratification factors were age (< 75 years; ≥ 75 years) and PaO_2_/F_I_O_2_ ratio (> 100 mmHg; ≤ 100 mmHg) at diagnosis of ARDS.

Patients in the invimestrocel group received standard treatment plus a single intravenous (IV) infusion of 9.0 × 10^8^ cells of invimestrocel administered at a rate of up to 10 mL/min over 30–60 min by free flow. Vials of invimestrocel provided by the sponsor were thawed and diluted into IV solution for infusion. Details of invimestrocel dose preparation and quality assurance are summarized in Additional file 2. During the first 2 h after the start of invimestrocel administration, vital signs were measured. Administration of other investigational drugs, products, or devices and short-term high-dose or pulse methylprednisolone therapy as the standard treatment were prohibited. Study period was the time from informed consent to day 180 of follow-up. Discontinuation criteria were patient withdrawal, discontinuation of the study due to an adverse event (AE), or investigator or sponsor decision.

### Outcome measures

Primary endpoint was the number of days of survival free from mechanical ventilation (VFDs) during the first 28 days after administration of study treatment. VFDs were calculated based on the time and date of extubation and re-intubation; one VFD was defined as a day free from the ventilator for 24 h. If death occurred before day 28 after administration, or mechanical ventilation continued after day 28, then VFDs were zero. If mechanical ventilation was discontinued before day 28, with X as the cumulative number of days with mechanical ventilation, then VFD was equal to 28 minus X. For patients who discontinued within 28 days for reasons other than death, VFDs were calculated assuming that a patient was on a ventilator on the day of discontinuation and onwards. Secondary efficacy endpoints included ventilator weaning rate on day 28, re-intubation rate, mortality on days 28, 60, 90, and 180 after treatment administration, and progression of chest imaging findings (radiographic and HRCT changes) through day 90 (obtained at screening, once between the time of ventilator weaning and discharge from hospital, and on days 14, 60, and 90). Procedures for confirmation and implementation of ventilator weaning are provided in Additional file 2.

Exploratory endpoints included parameters of inflammation and lung injury and the EuroQol 5-Dimension 5-Level (EQ-5D-5L) [30] QoL survey. Parameters of inflammation and lung injury included white blood cell count, neutrophil count, blood C-reactive protein (CRP), and lactate dehydrogenase. Inflammatory biomarkers included C-X-C motif chemokine 10 (CXCL10), interleukin-1β (IL-1β), interleukin-1 receptor type II (IL-1R2), interleukin-1 receptor antagonist (IL-1RA), interleukin-6 (IL-6), interleukin-8 (IL-8), interleukin-10 (IL-10), interleukin-12 (IL-12), sialylated carbohydrate antigen KL-6 (KL-6), matrix metalloproteinase-2 (MMP-2), programmed cell death-1 (PD-1), receptor for advanced glycation end products (RAGE), regulated on activation, normal T cell expressed and secreted (RANTES), surfactant protein-D (SP-D), transforming growth factor-β (TGF-β), and thrombospondin-1 (TSP-1). These were assessed at baseline (defined as the last measurement on day 0 pre-treatment or before [day –3 to –1]), and days 1, 2, 3, and 7. Biomarkers were quantified by quantitative enzyme-linked immunosorbent assay (ELISA) with the following kits: KL-6 (Stacia CLEIA KL-6, Sekisui Medical Co., Ltd.); SP-D (SP-D kit "Yamasa", Yamasa Corporation); the following kits were from R&D Systems (Minneapolis, MN, USA): TGF-β (Human TGF-β1 Immunoassay 2nd Generation), IL-1β (Human IL-1 beta/IL-1F2 Quantikine HS ELISA Kit), IL-6 (Quanti Glo ELISA Human IL-6 Immunoassay), IL-10 (Quantikine HS ELISA Human IL-10), IL-12 (Quantikine HS Human IL-12 Immunoassay), CXCL10 (Human CXCL10/IP-10 Quantikine ELISA Kit, #DIP100), IL-1R2 (Human IL-1 RII Quantikine ELISA Kit, #DR1B00), IL-1RA (Human IL-1ra/IL-1F3 Quantikine ELISA Kit, #DRA00B), MMP-2 (Total MMP-2 Quantikine ELISA Kit, #MMP200), RAGE (Human RAGE Quantikine ELISA Kit, #DRG00), RANTES (Human CCL5/RANTES Quantikine ELISA Kit, #DRN00B), and TSP-1 (Human Thrombospondin-1 Quantikine ELISA Kit, # DTSP10). Kits for IL-8 (Human IL-8 ELISA kit) and PD-1 (Human PD-1 ELISA Kit, #BMS2214) were from Thermo Fisher Scientific (Waltham, MA, USA). The self-reported EQ-5D-5L scale questionnaire was implemented to evaluate the patients’ health. Five dimensions of health are measured on a scale of 1–5: mobility, self-care, usual activities, pain and discomfort, and anxiety and depression at ventilator weaning, at discharge, and on days 60, 90, and 180 after administration.

Safety endpoints included AEs from informed consent to day 180 of follow-up or discontinuation. AE severity was assessed by the investigator as mild (sign or symptom easily tolerable and not interfering with daily life activities), moderate (significant discomfort and interfering with daily life activities), or severe (incapacitating or significantly interfering with daily life activities); infusion-related AEs were graded according to the Common Terminology Criteria for Adverse Events v4.0. Treatment-emergent AEs (TEAEs) were summarized by preferred term according to the Medical Dictionary for Regulatory Activities/Japanese edition Version 24.1. Any causal relationship was determined by the attending physician. Vital signs were collected throughout the study period at screening, 1 h (± 15 min) before, at the time of (0 min), and every 15 min (± 5 min) until 2 h after the start of administration, 3 h (± 15 min) and 4 h (± 15 min) after the start of administration, twice daily from day 1 until ventilator weaning, once daily from ventilator weaning until discharge, and if possible, on days 60, 90, and 180 after discharge. Laboratory test values were collected, including hematology, blood chemistry, and urinalysis, at screening, on days 1, 2, 3, and 7, and then every 7 days until discharge, at ventilator weaning, at discharge, and on days 60, 90, and 180 when possible.

### Statistical analysis

The target sample size was 30 patients (invimestrocel group: N = 20; standard group: N = 10) based on feasibility and not statistical power.

The modified intent-to-treat (mITT) analysis set included all randomized patients with at least one efficacy endpoint. The safety analysis set (SAS) included all randomized patients. Efficacy and safety analyses were performed on the mITT set and SAS, respectively. For continuous data, summary statistics were calculated; for categorical data, frequencies are presented. For the primary endpoint, means with 95% confidence intervals (CIs) are presented for each treatment group and an analysis of covariance was performed with treatment group, age at enrollment (< 75 years, ≥ 75 years), PaO_2_/F_I_O_2_ ratio (> 100 mmHg, ≤ 100 mmHg), and APACHE II score as covariates. Least squares (LS) means in each treatment group and difference in LS means between treatment groups with two-sided 95% CIs were estimated. For mortality, overall survival times were estimated by the Kaplan–Meier method and plotted; survival curves were compared with the log rank test. For calculation of VFDs, patients who discontinued for reasons other than death before day 28 were deemed to have used the ventilator on all the days after discontinuation; for all other endpoints, no imputation for missing data was performed. Outliers were included in analyses, and no adjustment of multiplicity was planned. For safety, counts of patients with events and frequencies of events are presented. Statistical analyses were performed using SAS 9.2 or later (SAS Institute Japan Ltd., Tokyo, Japan).

## Results

### Patient disposition

**Fig. 1:**
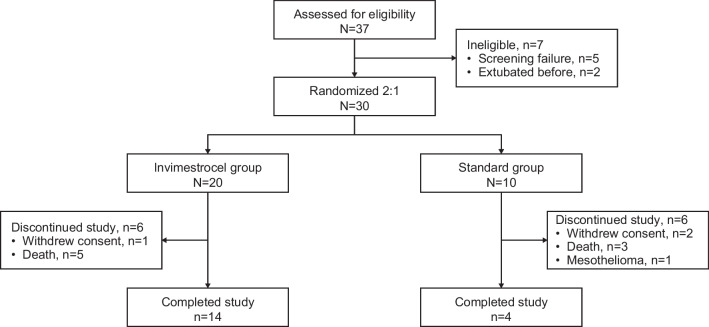
Patient disposition in the ONE-BRIDGE study

The study enrollment period was April 19, 2019–March 1, 2021 across 29 medical centers in Japan. Overall, 37 patients were enrolled and provided written informed consent; 30 patients met the eligibility criteria and were randomized to the invimestrocel group (N = 20) and standard group (N = 10). All 30 patients received treatment (Fig. 1). At data cutoff (September 24, 2021), 18 patients had completed the study; 12 patients had discontinued because of death (n = 8), withdrawn consent (n = 3), and mesothelioma (n = 1).

### Baseline patient demographics and clinical characteristics

Baseline patient demographics and clinical characteristics were generally balanced between treatment groups (Table 1). Median (interquartile range [IQR]) estimated HRCT score at screening was 230.0 (210.0–270.0) and 250.0 (210.0–270.0) and median (IQR) APACHE II score was 20.5 (17.0–24.0) and 20.0 (18.0–21.0) for the invimestrocel and standard groups, respectively.

**Table 1:** Baseline patient demographic and clinical characteristics

Characteristics	Invimestrocel group (N = 20)	Standard group (N = 10)
Age at informed consent obtained (years)		
Mean (SD)	69.2 (13.2)	66.5 (10.8)
Median	70.5	67.0
Min–max	44–89	48–85
Age category, n (%)		
< 75 years	13 (65)	7 (70)
≥ 75 years	7 (35)	3 (30)
Sex, n (%)		
Male	16 (80)	10 (100)
Female	4 (20)	0
Smoking status, n (%)		
Current	5 (25)	3 (30)
Former	9 (45)	5 (50)
Never	6 (30)	1 (10)
Missing, n	0	1
Alcohol consumption, n (%)		
Current	10 (50)	4 (40)
Former	8 (40)	4 (40)
Never	2 (10)	1 (10)
Missing, n	0	1
PaO_2_/F_I_O_2_ ratio at diagnosis (mmHg)		
Mean (SD)	125.7 (52.3)	151.3 (62.7)
Median (IQR)	117.1 (95.3–149.0)	128.0 (104.4–186.3)
PaO_2_/F_I_O_2_ ratio category, n (%)		
≤ 100 mmHg	6 (30)	2 (20)
100 mmHg to ≤ 200 mmHg	13 (65)	6 (60)
200 mmHg to ≤ 300 mmHg	1 (5)	2 (20)
Estimated HRCT score at screening (Evaluator 1)		
n	20	10
Mean (SD)	237.5 (43.0)	241.0 (35.7)
Median (IQR)	230.0 (210.0–270.0)	250.0 (210.0–270.0)
APACHE II score		
Mean (SD)	19.8 (5.1)	19.6 (3.0)
Median (IQR)	20.5 (17.0–24.0)	20.0 (18.0–21.0)

*APACHE II* Acute Physiology and Chronic Health Evaluation II, *HRCT* high-resolution computed tomography, *IQR* interquartile range, *max* maximum, *min* minimum, *PaO*_*2*_*/F*_*I*_*O*_*2*_ partial pressure arterial oxygen/fraction of inspired oxygen

### Primary outcome measure

The primary outcome measure of VFDs during the first 28 days after administration of study treatment did not statistically significantly differ between the invimestrocel group and the standard group (LS means [95% CI]: invimestrocel group, 11.6 [6.9–16.3]; standard group, 6.2 [− 0.4 to 12.8]; LS mean difference [95% CI] was 5.4 [− 1.9 to 12.8]; *p* = 0.1397) (Table 2). Median (IQR) VFDs during the first 28 days were 20.0 (0.0–24.0) in the invimestrocel group and 11.0 (0.0–14.0) in the standard group (Table 2).

**Table 2:** Ventilator-free days, ventilator weaning, re-intubation rate, and mortality rate (modified intent-to-treat analysis set)

	Invimestrocel group (N = 20)	Standard group (N = 10)	*p* value
Primary outcome			
VFD from day 0 to day 28, days			
Mean (SD)	14.8 (11.0)	10.6 (10.0)	
Median (IQR)	20.0 (0.0–24.0)	11.0 (0.0–14.0)	
LS mean^a^ (95% CI)	11.6 (6.9–16.3)	6.2 (–0.4 to 12.8)	
LS mean difference (95% CI)	5.4 (–1.9 to 12.8)		0.1397^b^
Secondary outcomes			
Ventilator weaning at day 28			
n (%)	13 (65)	3 (30)	−
Missing, n	5	5	
Number of patients with ventilation weaning at least once	15	9	
Re-intubation	4/15 (27)	3/9 (33)	−
Ventilation weaning after re-intubation	4/15 (27)	1/9 (11)	−
Mortality			
Day 28			
n/non-missing (%)	4/19 (21)	2/7 (29)	1.000^c^
Missing, n	1	3	
Day 60			
n/non-missing (%)	5/19 (26)	3/7 (43)	0.6353^c^
Missing, n	1	3	
Day 90			
n/non-missing (%)	5/19 (26)	3/7 (43)	0.6353^c^
Missing, n	1	3	
Day 180^d^			
n/non-missing (%)	5/19 (26)	3/7 (43)	0.6353^c^
Missing, n	1	3	

Percentages are calculated using the N as the denominator except where indicated (invimestrocel group, N = 20; standard group, N = 10)

*APACHE II* Acute Physiology and Chronic Health Evaluation II, *IQR* interquartile range, *LS* least squares, *PaO*_*2*_*/F*_*I*_*O*_*2*_ partial pressure arterial oxygen/fraction of inspired oxygen, *VFD* ventilator-free days

^a^Analysis of covariance using treatment group, age at enrollment (< 75 years, ≥ 75 years), PaO_2_/F_I_O_2_ ratio (> 100 mmHg, ≤ 100 mmHg), and APACHE II score as covariates

^b^Wilcoxon rank sum test

^c^Fisher’s exact test

^d^Those who completed the study before day 180 were considered alive

### Secondary and exploratory outcome measures

Ventilator weaning rate at day 28 was 65% (13/20) and 30% (3/10) for the invimestrocel and standard groups, respectively (Table 2). Re-intubation rate was 27% (4/15) and 33% (3/9) and the ventilator weaning rate after re-intubation was 27% (4/15) and 11% (1/9) in the invimestrocel and standard groups, respectively.

**Fig. 2:**
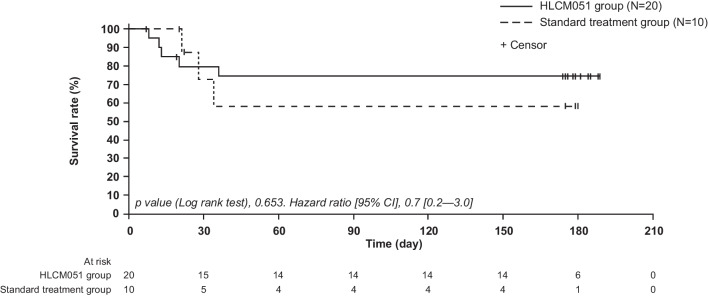
Kaplan–Meier plot of overall survival (modified intent-to-treat analysis set)

Mortality rates through day 180 were numerically lower for the invimestrocel group versus the standard group at all time points but were not statistically significantly different between the groups (Table 2). The Kaplan–Meier survival curves for the two groups were not statistically significantly different (hazard ratio [95% CI] 0.7 [0.2–3.0]; *p* = 0.653; Fig. 2); median survival was not reached in either group.

**Fig. 3:**
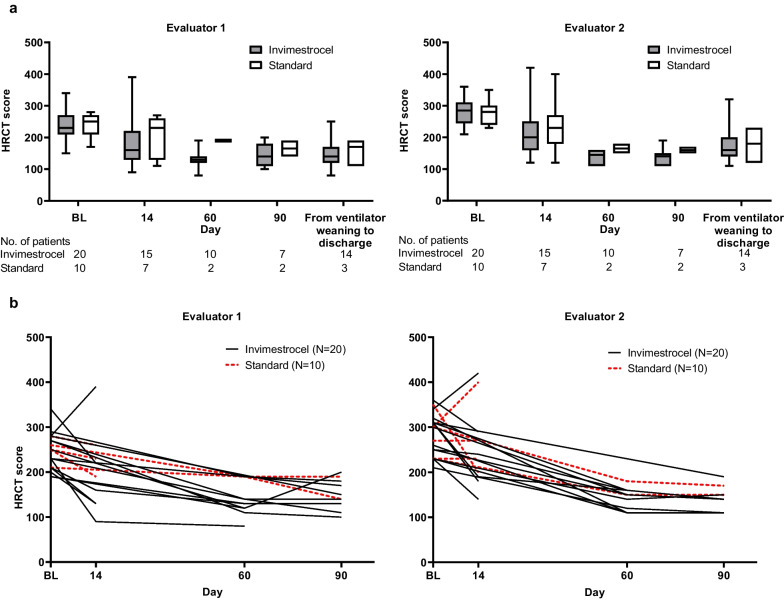
Time course of HRCT score (modified intent-to-treat analysis set). **a** HRCT score by evaluator. Data are presented as median with upper and lower quartiles. **b** Individual patient HRCT scores by evaluator. Discharge data are not shown. Baseline was defined as the last measurement at screening. *ARDS* acute respiratory distress syndrome, *BL* baseline, *HRCT* high-resolution computed tomography

Median HRCT score through day 90, and from ventilator weaning to discharge, was numerically lower in the invimestrocel group versus the standard group at most time points but overlap of the scores between the groups and a similar trend over time for both groups were observed (Fig. 3).

**Fig. 4:**
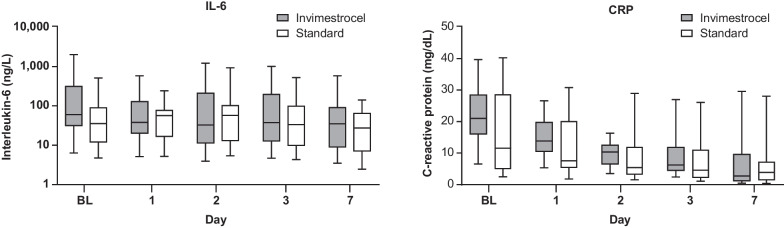
**a** Interleukin-6 and **b** C-reactive protein plasma concentrations over time (modified intent-to-treat analysis set). Data are presented as medians with upper and lower quartiles. Baseline is defined as the last measurement on day 0 pre-treatment or before (day − 3 to − 1). *ARDS* acute respiratory distress syndrome, *BL* baseline, *CRP* C-reactive protein, *IL-6* interleukin-6

Median (IQR) change in IL-6 levels from baseline to day 7 was similar between the groups (invimestrocel group, − 10.96 [− 213.92 to − 0.92] ng/L; standard group, − 10.83 [− 86.7 to 59.66] ng/L), and a trend for decreased CRP levels was observed in both groups (median [IQR]: invimestrocel group, − 13.8 [− 24.95 to − 5.46]; standard group, − 5.3 [− 20.18 to − 1.57] mg/dL; Fig. 4). No meaningful changes in other biomarkers were observed (Additional file 1: Table S2).

**Fig. 5:**
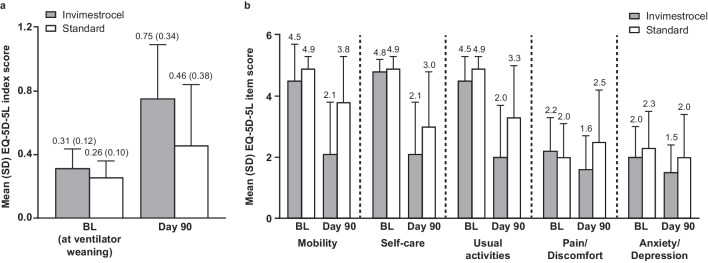
Patient quality of life in the invimestrocel and standard treatment groups. (**a)** EQ-5D-5L index score and (**b**) summary score of each item of EQ-5D-5L at baseline and day 90 after treatment administration. Baseline was defined as ventilator weaning. *ARDS* acute respiratory distress syndrome, *BL* baseline, *EQ-5D-5L* EuroQol 5-Dimension 5-Level

The EQ-5D-5L index score at day 90 indicated that the health of surviving patients in the invimestrocel group was better than that of the standard group (0.75 [0.34] vs 0.46 [0.38], respectively; Fig. 5a). An improvement in each EQ-5D-5L item at day 90 versus baseline was observed in both treatment groups (Fig. 5b).

### Safety

All patients in both groups reported ≥ 1 TEAE (Table 3); 10 (50%) patients in the invimestrocel group and 4 (40%) patients in the standard group experienced ≥ 1 serious TEAE. Five (25%) patients experienced TEAEs possibly related to invimestrocel during the study; events were arrhythmia, atrial fibrillation, increased sputum, abnormal hepatic function, pyrexia, increased blood pressure, increased pancreatic enzymes (all one patient each), and chills (two patients; one event classified as a serious TEAE that resolved quickly). At data cutoff, all possibly related TEAEs had resolved or were resolving. TEAEs leading to death were sepsis, systemic candida, and ARDS (one patient each) in the invimestrocel group, and multiple organ dysfunction syndrome (one patient) in the standard group. TEAEs leading to death were all unrelated to invimestrocel.

**Table 3:** Treatment-emergent adverse events by preferred term (Safety analysis set)

Variable	Invimestrocel group (N = 20)	Standard treatment group (N = 10)
≥ 1 TEAE	20 (100)	10 (100)
≥ 1 serious TEAE	10 (50)	4 (40)
≥ 1 TEAE leading to death	3 (15)	1 (10)
≥ 1 TEAE possibly related to invimestrocel	5 (25)	0
≥ 1 serious TEAE possibly related to invimestrocel	1 (5)	0
TEAEs occurring in ≥ 2 patients in the invimestrocel groups		
Insomnia	9 (45)	5 (50)
Diarrhea	7 (35)	1 (10)
Constipation	6 (30)	3 (30)
Pyrexia	6 (30)	0
Anemia	5 (25)	0
Decubitus ulcer	4 (20)	2 (20)
Hepatic function abnormal	4 (20)	1 (10)
Pneumonia	3 (15)	1 (10)
Atrial fibrillation	3 (15)	1 (10)
Acute kidney injury	3 (15)	1 (10)
Acute respiratory distress syndrome	2 (10)	1 (10)
Hypernatremia	2 (10)	2 (20)
Atelectasis	2 (10)	0
Cough	2 (10)	1 (10)
Pneumothorax	2 (10)	0
Skin exfoliation	2 (10)	0
Drug eruption	1 (5)	1 (10)
Arthralgia	2 (10)	0
Chills	2 (10)	0
ALT increased	2 (10)	0
AST increased	2 (10)	0

Data are n (%)

*ALT* alanine transaminase, *AST* aspartate transaminase, *TEAE* treatment-emergent adverse event

## Discussion

This is the first study to evaluate the efficacy and safety of multipotent adult progenitor cells (invimestrocel) in a clearly defined set of patients with ARDS. In this study, invimestrocel was evaluated in patients with ARDS caused by pneumonia and with severity defined by the APACHE II score and HRCT score. Patients who were likely to die of severe systemic organ failure within a few days were specifically excluded by an APACHE II score ≥ 27, while those at a high risk of progressive pulmonary fibroproliferation based on an HRCT score ≥ 211, which is considered optimal for examining the repair effect of MSCs on injured lungs, were included [5, 26, 27]. In this open-label phase 2 study of Japanese patients, the number of VFDs during the first 28 days after administration of study treatment was numerically higher and mortality rates through day 180 were numerically lower in the invimestrocel group versus the standard group but the differences were not statistically significantly different between the groups. There was no apparent difference in patient background characteristics between patients in the invimestrocel group and the standard group, and any effect of patient background characteristics on the results is expected to be small. Five (25%) patients experienced TEAEs that were assessed by investigators to be possibly related to invimestrocel; one event of chills that was classified as serious resolved quickly. Overall, invimestrocel was well tolerated in Japanese patients with ARDS caused by pneumonia. A confirmatory study with a diverse patient population is warranted to further evaluate invimestrocel as a potential new treatment that may improve the prognosis of ARDS.

Although corticosteroids have been shown to improve survival in patients with ARDS caused by COVID-19 [31], no effective medication for classical ARDS that significantly improves survival has been identified. In a trial of patients with moderate-to-severe ARDS using IV interferon β-1a, no significant difference in mortality rates at day 28 was observed with treatment compared with placebo [32]. In a trial using IV high-dose vitamin C compared with placebo, a significant result in mortality rates at day 28 was observed; however, the authors hypothesized that this result may have represented the effects of vitamin C on underlying sepsis-induced biological abnormalities [33]. Although significant improvements in patient survival with umbilical cord MSC treatment compared with standard of care were observed in two studies in patients with COVID-19 and ARDS [34, 35], MSC treatment did not improve the 28-day mortality rate versus placebo [13]. In the current study, mortality rates were numerically lower with invimestrocel versus standard treatment from day 28 and up to day 180 after treatment (21–26% vs 29–43%, respectively). These results, although not statistically significant, reaffirm the improved day 28 mortality rates observed in the MUST-ARDS study of invimestrocel versus placebo in patients with moderate-to-severe ARDS (25% vs 40%, respectively) [23]. Overall, these consistent results suggest that invimestrocel may offer a promising new treatment option to add to the current standard of care for the treatment of ARDS, and that cell therapy could be effective in patients at high risk of pulmonary fibroproliferation associated with ARDS, which affects a patient’s long-term prognosis for pneumonia-induced ARDS.

The number of TEAEs was similar between the invimestrocel and standard treatment groups, and the number of TEAEs possibly related to invimestrocel was small, with all events being resolved or resolving at the time of data cutoff. The safety profile observed in this study aligns with that of the MUST-ARDS trial, in which pyrexia was the only possibly related TEAE observed in patients treated with invimestrocel [23]. The results of this study and others investigating the safety of MSC treatment have shown that cell treatments can be safely administered to patients with ARDS [13, 15, 35–37].

The limitations of this study are that it was open-label, the number of patients was small (especially in the standard group), and statistically significant differences could not be determined. An open-label, comparator-controlled study design using standard treatment as a control was chosen because the administration of placebo may be associated with a risk of aggravation of the disease due to fluid loading. Although the study was open-label, quality was enhanced by the use of a predefined ventilator weaning protocol with confirmation by physicians that extubation proceeded without concerns. In addition, physician discretion was minimized where possible, e.g., by using multiple evaluators for HRCT score, and a third evaluator was included in ventilator weaning to confirm the appropriateness of weaning and whether extubation was not arbitrarily delayed or hastened. Furthermore, HRCT score used as part of the patient selection in this study is validated only in the Japanese population, which limits the generalizability of the study. We included patients with high HRCT scores to select patients with ARDS at a high risk of progressive pulmonary fibroproliferation, and we specifically excluded patients at risk of early death using APACHE II scores; however, these criteria will make it more difficult to directly compare our data with other clinical studies.

## Conclusions

In this phase 2 study of Japanese patients with ARDS caused by pneumonia, treatment with invimestrocel plus standard treatment compared with standard treatment alone resulted in no significant difference in the number of VFDs over 28 days but may result in improved survival. Further studies in blinded, placebo-controlled trials are required to further evaluate early withdrawal from the ventilator and reduced mortality with the addition of invimestrocel to standard treatment.

### Supplementary Information

**Additional file 1**. **Table S1**: Study sites and institutional review boards. **Table S2**: Summary of laboratory values for inflammation or lung injury parameters at baseline and on days 1, 2, 3, and 7 (modified intent-to-treat analysis set).

**Additional file 2**. Methods.

## Data Availability

The datasets generated and analysed during the current study are not publicly available due to patient confidentiality but may be available from the corresponding author on reasonable request.

## Abbreviations

AE: Adverse event
APACHE: Acute Physiology and Chronic Health Evaluation
ARDS: Acute respiratory distress syndrome
CI: Confidence interval
CRP: C-reactive protein
EQ-5D-5L: EuroQol 5-dimension 5-level
HRCT: High-resolution computed tomography
IQR: Interquartile range
IV: Intravenous
LS: Least squares
mITT: Modified intent-to-treat
MSC: Mesenchymal stem cells
PaO_2_/F_I_O_2_: Partial pressure arterial oxygen/fraction of inspired oxygen
QoL: Quality of life
SAS: Safety analysis set
TEAE: Treatment-emergent adverse event
VFD: Ventilator-free day
