# Supplementary material for: Clinical efficacy and safety of multipotent adult progenitor cells (invimestrocel) for acute respiratory distress syndrome (ARDS) caused by pneumonia: a randomized, open-label, standard therapy–controlled, phase 2 multicenter study (ONE-BRIDGE)
Source: Stem Cell Res Ther. 2023 Aug 22;14:217. doi: 10.1186/s13287-023-03451-z (PMC10464414; doi:10.1186/s13287-023-03451-z)
Supplement: Supplementary file 1 — Additional file 1. Table S1: Study sites and institutional review boards. Table S2: Summary of laboratory values for inflammation or lung injury parameters at baseline and on days 1, 2, 3, and 7 (modified intent-to-treat analysis set). [file 13287_2023_3451_MOESM1_ESM.docx]

**Additional file 1**

**Table S1.**

**Study sites and institutional review boards**

| **Study site number** | **Study site** | **Institutional review board** | **Approval date** | **Approval Number** |
| --- | --- | --- | --- | --- |
| 1 | Saiseikai Kumamoto Hospital | Hospital’s IRB | 13 December 2018 | NA^a^ |
| 2 | University Hospital, Kyoto Prefectural University of Medicine | Hospital’s IRB | 10 January 2019 | 2018-022 |
| 3 | Hirosaki University Hospital | Hospital’s IRB | 12 December 2018 | 再4 |
| 4 | Showa University Hospital | Hospital’s IRB | 29 January 2019 | 1811014 |
| 5 | Tosei General Hospital | Hospital’s IRB | 14 February 2019 | H310102Multi |
| 6 | The Jikei University Hospital | Hospital’s IRB | 27 February 2019 | 再30‐2（3） |
| 7 | Aso Iizuka Hospital | Hospital’s IRB | 7 January 2019 | 30-11 |
| 8 | Keio University Hospital | Hospital’s IRB | 24 January 2019 | 18-033 |
| 9 | Nihon University Itabashi Hospital | Central review board for all Nihon University hospitals | 26 February 2019 | Z1902-084 |
| 10 | Kobe City Medical Center General Hospital | Center’s IRB | 28 December 2018 | 治18-27 |
| 11 | The Hokkaido Medical Center | Center’s IRB | 6 March 2019 | 1903-A-07 |
| 12 | Hiroshima University Hospital | Hospital’s IRB | 9 May 2019 | 70004 |
| 13 | Kobe City Medical Center West Hospital | Center’s IRB | 11 January 2019 | 2018-002 |
| 14 | Shimane University Hospital | Hospital’s IRB | 7 March 2019 | 2019-2 |
| 15 | Nagasaki University Hospital | Hospital’s IRB | 29 January 2019 | H30-301 |
| 16 | Saga University Hospital | Hospital’s IRB | 4 February 2019 | 2018-11-01 |
| 17 | Takarazuka City Hospital | Medical Corporation Toukeikai Kitamachi Clinic’s IRB | 19 February 2019 | NA^a^ |
| 18 | Nara Medical University Hospital | Hospital’s IRB | 19 March 2019 | 18-018 |
| 19 | Ogaki Municipal Hospital | Hospital’s IRB | 28 February 2019 | NA^a^ |
| 20 | Fujita Health Medical University Hospital | Hospital’s IRB | 22 May 2019 | 再生-1 |
| 21 | St. Luke’s International Hospital | Hospital’s IRB | 17 October 2019 | 19-G14 |
| 22 | Shiga University of Medical Science Hospital | Hospital’s IRB | 2 October 2019 | 19-12 |
| 23 | Tokyo Medical University Hospital | Hospital’s IRB | 21 October 2019 | C2019-019 |
| 24 | Tokyo Medical and Dental University Hospital | Hospital’s IRB | 28 October 2019 | 2019-0021 |
| 25 | Yokohama City University Hospital | Hospital’s IRB | 19 December 2019 | 19-515 |
| 26 | Osaka University Hospital | Hospital’s IRB | 13 December 2019 | 192002-B |
| 27 | Nagoya City University Hospital | Hospital’s ethics committees | 15 November 2019 | 13-19-0002 |
| 28 | Nagoya University Hospital | Hospital’s IRB | 6 January 2020 | 310029 |
| 29 | Yokohama City University Medical Center | Center’s IRB | 10 January 2020 | 119-018 |

^a^Approval number was not provided by the IRB.
*IRB* institutional review board, *NA* not available

**Table S2.**

**Summary of laboratory values for inflammation or lung injury parameters at baseline and on days 1, 2, 3, and 7 (modified intent-to-treat analysis set)**

| **Invimestrocel group (N=20)** | | | | | |
| --- | --- | --- | --- | --- | --- |
| **Parameter** | **Baseline^a^** | **Day 1** | **Day 2** | **Day 3** | **Day 7** |
| **White blood cell count (10^3^/uL)** |  |  |  |  |  |
| n | 20 | 20 | 20 | 20 | 19 |
| Mean | 14.496 | 14.165 | 12.815 | 12.488 | 14.767 |
| SD | 7.910 | 7.897 | 8.581 | 7.323 | 6.418 |
| Q1 | 6.65 | 7.375 | 6.925 | 6.575 | 9.75 |
| Median | 14.095 | 12.85 | 10.85 | 10.65 | 14.28 |
| Q3 | 20.05 | 18.08 | 14.8 | 18.15 | 20 |
| **Neutrophils/leukocytes (fraction of 1)** |  |  |  |  |  |
| n | 20 | 20 | 20 | 20 | 19 |
| Mean | 0.89745 | 0.89565 | 0.86950 | 0.85125 | 0.88705 |
| SD | 0.04970 | 0.05905 | 0.05846 | 0.06175 | 0.05366 |
| Q1 | 0.86975 | 0.86425 | 0.8315 | 0.808 | 0.843 |
| Median | 0.9035 | 0.908 | 0.885 | 0.845 | 0.89 |
| Q3 | 0.93075 | 0.937 | 0.9055 | 0.8985 | 0.9285 |
| **C-reactive protein (mg/dL)** |  |  |  |  |  |
| n | 20 | 20 | 20 | 20 | 19 |
| Mean | 21.775 | 14.952 | 9.906 | 8.744 | 6.696 |
| SD | 9.485 | 6.150 | 4.020 | 6.589 | 7.225 |
| Q1 | 16.08 | 10.56 | 6.5725 | 4.545 | 1.23 |
| Median | 21.04 | 13.84 | 10.33 | 6.24 | 2.71 |
| Q3 | 28.435 | 19.7725 | 12.525 | 11.81 | 9.59 |
| **Lactate dehydrogenase (U/L)** |  |  |  |  |  |
| n | 20 | 20 | 20 | 20 | 20 |
| Mean | 410.6 | 440.9 | 426.0 | 381.5 | 398.4 |
| SD | 253.2 | 262.6 | 254.7 | 210.2 | 325.2 |
| Q1 | 231.25 | 263.5 | 269.75 | 257.75 | 261.5 |
| Median | 296 | 343 | 350 | 320 | 311 |
| Q3 | 520.75 | 499.5 | 452.75 | 406.25 | 381.75 |
| **Chemokine (C-X-C Motif) ligand 10 (ng/L)** |  |  |  |  |  |
| n | 20 | 20 | 20 | 20 | 19 |
| Mean | 758.89 | 749.77 | 503.89 | 402.84 | 455.09 |
| SD | 1443.18 | 1398.44 | 807.29 | 519.55 | 346.15 |
| Q1 | 91.15 | 109.5 | 158.5 | 129.75 | 163 |
| Median | 189 | 236.5 | 201 | 209.5 | 379 |
| Q3 | 422.75 | 357.75 | 398.5 | 379 | 787.5 |
| **Interleukin-1β (ng/L)** |  |  |  |  |  |
| n | 20 | 20 | 20 | 20 | 19 |
| Mean | 0.2250 | 0.2189 | 0.2963 | 0.3401 | 0.3755 |
| SD | 0.1936 | 0.1995 | 0.2471 | 0.4390 | 0.3389 |
| Q1 | 0.125 | 0.125 | 0.125 | 0.125 | 0.125 |
| Median | 0.125 | 0.125 | 0.1445 | 0.125 | 0.161 |
| Q3 | 0.2295 | 0.2005 | 0.454 | 0.321 | 0.656 |
| **Interleukin-1 receptor type II (ng/L)** |  |  |  |  |  |
| n | 20 | 20 | 20 | 20 | 19 |
| Mean | 61794.1 | 69852.7 | 69962.7 | 62383.4 | 37501.8 |
| SD | 107552.3 | 111066.8 | 112992.3 | 107866.1 | 54297.0 |
| Q1 | 19711.25 | 26876.75 | 24858.5 | 22523.5 | 18913 |
| Median | 26822 | 30108.5 | 31750.5 | 28519 | 24425 |
| Q3 | 52008.5 | 57591.75 | 48316.5 | 41361.5 | 31497.5 |
| **Interleukin-1 receptor antagonist (ng/L)** |  |  |  |  |  |
| n | 20 | 20 | 20 | 20 | 19 |
| Mean | 3121.0 | 2600.5 | 2030.1 | 1878.6 | 1915.3 |
| SD | 3397.2 | 3229.0 | 1363.6 | 1712.5 | 1345.1 |
| Q1 | 1057.5 | 967.75 | 995.75 | 749 | 944 |
| Median | 1590 | 1580 | 1785 | 1210 | 1450 |
| Q3 | 4022.5 | 2707.5 | 2767.5 | 2005 | 2855 |
| **Interleukin-6 (ng/L)** |  |  |  |  |  |
| n | 20 | 20 | 20 | 20 | 19 |
| Mean | 266.106 | 107.614 | 157.508 | 153.609 | 98.744 |
| SD | 464.784 | 142.009 | 277.718 | 258.542 | 159.164 |
| Q1 | 31.35 | 20.15 | 11.3575 | 12.8 | 9.03 |
| Median | 59.65 | 38 | 32.7 | 37.5 | 34.8 |
| Q3 | 313.75 | 127.75 | 209.75 | 194 | 90.55 |
| **Interleukin-8 (ng/L)** |  |  |  |  |  |
| n | 20 | 20 | 20 | 20 | 19 |
| Mean | 35.80 | 24.84 | 22.02 | 20.61 | 18.83 |
| SD | 54.79 | 30.38 | 23.22 | 17.65 | 13.83 |
| Q1 | 10.25 | 8.775 | 8.25 | 8.225 | 8 |
| Median | 15.1 | 16.95 | 16.15 | 16.05 | 11.8 |
| Q3 | 26.8 | 29.65 | 23.025 | 23.175 | 26 |
| **Interleukin-10 (ng/L)** |  |  |  |  |  |
| n | 20 | 20 | 20 | 20 | 19 |
| Mean | 5.362 | 3.176 | 3.495 | 11.079 | 4.523 |
| SD | 6.941 | 3.053 | 2.545 | 38.616 | 3.505 |
| Q1 | 1.0375 | 1.1575 | 1.4325 | 1.395 | 1.475 |
| Median | 1.945 | 1.87 | 2.695 | 2.57 | 3.53 |
| Q3 | 6.2225 | 4.365 | 5.23 | 3.2975 | 6.895 |
| **Interleukin-12 (ng/L)** |  |  |  |  |  |
| n | 20 | 20 | 20 | 20 | 19 |
| Mean | 0.865 | 0.942 | 0.934 | 0.951 | 0.998 |
| SD | 0.303 | 0.516 | 0.479 | 0.539 | 0.561 |
| Q1 | 0.78 | 0.78 | 0.78 | 0.78 | 0.78 |
| Median | 0.78 | 0.78 | 0.78 | 0.78 | 0.78 |
| Q3 | 0.78 | 0.78 | 0.78 | 0.78 | 0.84 |
| **Sialylated carbohydrate antigen KL-6 (U/mL)** |  |  |  |  |  |
| n | 20 | 20 | 20 | 20 | 19 |
| Mean | 378.9 | 404.2 | 449.2 | 411.6 | 507.8 |
| SD | 365.4 | 428.4 | 573.3 | 442.8 | 897.9 |
| Q1 | 185.5 | 183.75 | 182.25 | 178.75 | 188.5 |
| Median | 218 | 209.5 | 225 | 230 | 212 |
| Q3 | 361.5 | 384.75 | 371.5 | 382 | 336 |
| **Matrix metalloproteinase-2 (ug/L)** |  |  |  |  |  |
| n | 20 | 20 | 20 | 20 | 19 |
| Mean | 204.25 | 212.55 | 218.05 | 214.87 | 231.84 |
| SD | 89.11 | 83.69 | 113.68 | 97.93 | 89.85 |
| Q1 | 156.25 | 165 | 150.25 | 147.5 | 161 |
| Median | 170.5 | 194 | 169.5 | 188.5 | 210 |
| Q3 | 219.5 | 236 | 256.25 | 258.5 | 296 |
| **Programmed cell death-1 (ng/L)** |  |  |  |  |  |
| n | 20 | 20 | 20 | 20 | 19 |
| Mean | 93.084 | 84.567 | 84.456 | 83.338 | 83.657 |
| SD | 161.089 | 160.651 | 161.282 | 160.930 | 163.795 |
| Q1 | 31.265 | 29.975 | 32.805 | 31.1225 | 30.19 |
| Median | 46.97 | 40.95 | 41.525 | 42.3 | 45.88 |
| Q3 | 87.76 | 59.4825 | 60.7625 | 58.9875 | 57.555 |
| **RAGE (ng/L)** |  |  |  |  |  |
| n | 20 | 20 | 20 | 20 | 19 |
| Mean | 3259.14 | 2751.72 | 2141.53 | 1877.22 | 1539.67 |
| SD | 1752.50 | 1486.09 | 1489.55 | 1443.46 | 1158.98 |
| Q1 | 1767.2 | 1603.875 | 1108.775 | 993.05 | 691.4 |
| Median | 2957.7 | 2401.85 | 1617.95 | 1254.05 | 1145.4 |
| Q3 | 4456.7 | 3967 | 3111.95 | 2492.3 | 2323.5 |
| **Chemokine ligand 5 (ng/L)** |  |  |  |  |  |
| n | 20 | 20 | 20 | 20 | 19 |
| Mean | 35511.5 | 34882.0 | 27971.5 | 31547.0 | 41174.7 |
| SD | 32520.5 | 31299.0 | 24768.2 | 29312.3 | 27526.1 |
| Q1 | 15675 | 14375 | 8947.5 | 11380 | 25100 |
| Median | 27350 | 25550 | 20500 | 25750 | 37600 |
| Q3 | 34775 | 45225 | 43500 | 37125 | 47800 |
| **SP-D (ug/L)** |  |  |  |  |  |
| n | 20 | 20 | 20 | 20 | 19 |
| Mean | 239.72 | 294.62 | 284.46 | 267.64 | 244.78 |
| SD | 199.48 | 238.33 | 193.24 | 182.63 | 472.55 |
| Q1 | 114.075 | 156.75 | 163.5 | 152 | 85.2 |
| Median | 169.5 | 198 | 242 | 252 | 123 |
| Q3 | 304 | 392.5 | 372.5 | 317.25 | 183 |
| **Transforming growth factor-β (ug/L)** |  |  |  |  |  |
| n | 20 | 20 | 20 | 20 | 19 |
| Mean | 3.027 | 3.125 | 3.139 | 2.884 | 3.610 |
| SD | 1.005 | 1.466 | 1.968 | 1.106 | 2.341 |
| Q1 | 2.335 | 2.155 | 2.2725 | 2.1175 | 2.27 |
| Median | 2.86 | 2.54 | 2.615 | 2.61 | 3 |
| Q3 | 3.5275 | 3.585 | 3.005 | 3.1575 | 3.62 |
| **Thrombospondin-1 (ug/L)** |  |  |  |  |  |
| n | 20 | 20 | 20 | 20 | 19 |
| Mean | 12704.5 | 12474.1 | 10140.0 | 13885.8 | 14027.4 |
| SD | 10644.5 | 10868.9 | 7586.9 | 11550.6 | 9604.1 |
| Q1 | 5307.5 | 5357.5 | 3475 | 5737.5 | 8875 |
| Median | 10170 | 9995 | 10240 | 8905 | 11300 |
| Q3 | 16175 | 18100 | 14475 | 17700 | 20300 |

| **Standard treatment group (N=10)** | | | | | |
| --- | --- | --- | --- | --- | --- |
| **Parameter** | **Baseline^a^** | **Day 1** | **Day 2** | **Day 3** | **Day 7** |
| **White blood cell count (10^3^/uL)** |  |  |  |  |  |
| n | 10 | 10 | 10 | 10 | 10 |
| Mean | 10.755 | 11.361 | 9.697 | 10.111 | 13.122 |
| SD | 4.856 | 5.536 | 2.958 | 4.041 | 4.902 |
| Q1 | 6.775 | 6.95 | 8.5 | 7.325 | 11.35 |
| Median | 8.9 | 9.65 | 9.55 | 9.96 | 12.55 |
| Q3 | 15.74 | 14.9 | 9.875 | 13.3 | 13.425 |
| **Neutrophils/leukocytes (fraction of 1)** |  |  |  |  |  |
| n | 10 | 10 | 9 | 10 | 10 |
| Mean | 0.86850 | 0.88410 | 0.84767 | 0.86780 | 0.82810 |
| SD | 0.10880 | 0.10054 | 0.08931 | 0.08519 | 0.10168 |
| Q1 | 0.81175 | 0.8305 | 0.8 | 0.833 | 0.76825 |
| Median | 0.9165 | 0.9305 | 0.85 | 0.8695 | 0.8505 |
| Q3 | 0.94825 | 0.943 | 0.907 | 0.9275 | 0.87575 |
| **C-reactive protein (mg/dL)** |  |  |  |  |  |
| n | 10 | 10 | 10 | 10 | 10 |
| Mean | 16.900 | 12.419 | 8.953 | 7.833 | 6.259 |
| SD | 13.639 | 10.733 | 8.582 | 8.028 | 8.173 |
| Q1 | 4.99225 | 5.31 | 3.1525 | 2.2175 | 1.469 |
| Median | 11.55 | 7.528 | 5.415 | 4.55 | 3.865 |
| Q3 | 28.615 | 20.0425 | 11.9725 | 11.03 | 7.18 |
| **Lactate dehydrogenase (u/l)** |  |  |  |  |  |
| n | 10 | 10 | 10 | 10 | 10 |
| Mean | 478.0 | 461.0 | 441.2 | 424.8 | 368.5 |
| SD | 255.4 | 210.2 | 198.4 | 180.2 | 127.3 |
| Q1 | 327.75 | 316.25 | 313.75 | 292.25 | 290.25 |
| Median | 382.5 | 376.5 | 377 | 381 | 336 |
| Q3 | 633 | 674.5 | 598 | 528.5 | 398 |
| **Chemokine (C-X-C Motif) ligand 10 (ng/L)** |  |  |  |  |  |
| n | 9 | 9 | 9 | 9 | 8 |
| Mean | 1956.58 | 1169.78 | 722.56 | 579.67 | 472.63 |
| SD | 3191.94 | 1372.46 | 610.10 | 403.15 | 414.60 |
| Q1 | 185 | 150 | 186 | 198 | 211.5 |
| Median | 752 | 800 | 847 | 602 | 380.5 |
| Q3 | 2050 | 1430 | 973 | 847 | 492.5 |
| **Interleukin-1β (ng/L)** |  |  |  |  |  |
| n | 10 | 10 | 10 | 10 | 9 |
| Mean | 0.2234 | 0.1522 | 0.2773 | 0.2327 | 0.2469 |
| SD | 0.2149 | 0.0503 | 0.4201 | 0.2416 | 0.1734 |
| Q1 | 0.125 | 0.125 | 0.125 | 0.125 | 0.125 |
| Median | 0.125 | 0.125 | 0.125 | 0.125 | 0.164 |
| Q3 | 0.19375 | 0.15575 | 0.1745 | 0.2205 | 0.328 |
| **Interleukin-1 receptor type II (ng/L)** |  |  |  |  |  |
| n | 9 | 9 | 9 | 9 | 8 |
| Mean | 81411.7 | 78980.2 | 62933.2 | 52995.2 | 31263.0 |
| SD | 110556.1 | 100285.5 | 64389.3 | 45771.0 | 7867.1 |
| Q1 | 32616 | 35120 | 32440 | 26926 | 27242.75 |
| Median | 45571 | 46116 | 36535 | 37090 | 34214.5 |
| Q3 | 53813 | 71336 | 72035 | 64519 | 37029.5 |
| **Interleukin-1 receptor antagonist (ng/L)** |  |  |  |  |  |
| n | 9 | 9 | 9 | 9 | 8 |
| Mean | 3055.2 | 2143.3 | 2748.1 | 2789.0 | 2085.9 |
| SD | 5044.0 | 1955.4 | 2804.2 | 3157.0 | 1702.7 |
| Q1 | 649 | 911 | 804 | 976 | 1182.5 |
| Median | 1250 | 1890 | 1480 | 1330 | 1340 |
| Q3 | 2550 | 2210 | 3840 | 2250 | 2092.5 |
| **Interleukin-6 (ng/L)** |  |  |  |  |  |
| n | 10 | 10 | 10 | 10 | 9 |
| Mean | 94.295 | 73.443 | 147.047 | 103.401 | 47.662 |
| SD | 154.354 | 76.731 | 280.248 | 162.798 | 53.114 |
| Q1 | 12.025 | 16.85 | 13.175 | 9.975 | 7.1 |
| Median | 35.2 | 56.7 | 57 | 33.5 | 27.5 |
| Q3 | 88.825 | 76.85 | 101.575 | 98.15 | 64.4 |
| **Interleukin-8 (ng/L)** |  |  |  |  |  |
| n | 10 | 10 | 10 | 10 | 9 |
| Mean | 24.31 | 22.30 | 20.65 | 21.63 | 13.33 |
| SD | 20.07 | 10.86 | 15.02 | 20.05 | 6.65 |
| Q1 | 13.575 | 16.05 | 11.8 | 13.3 | 8 |
| Median | 18.5 | 20.8 | 15 | 14.55 | 11 |
| Q3 | 24.65 | 30.6 | 21.275 | 21.975 | 17.6 |
| **Interleukin-10 (ng/L)** |  |  |  |  |  |
| n | 10 | 10 | 10 | 10 | 9 |
| Mean | 5.191 | 5.458 | 7.067 | 6.222 | 3.796 |
| SD | 6.355 | 9.159 | 13.617 | 11.309 | 2.874 |
| Q1 | 1.33 | 1.3675 | 2 | 1.8925 | 1.54 |
| Median | 1.785 | 2.55 | 2.795 | 2.74 | 3.35 |
| Q3 | 5.2775 | 4.4575 | 4.025 | 3.47 | 5.82 |
| **Interleukin-12 (ng/L)** |  |  |  |  |  |
| n | 10 | 10 | 10 | 10 | 9 |
| Mean | 0.780 | 0.780 | 0.821 | 0.818 | 0.816 |
| SD | 0.000 | 0.000 | 0.130 | 0.120 | 0.107 |
| Q1 | 0.78 | 0.78 | 0.78 | 0.78 | 0.78 |
| Median | 0.78 | 0.78 | 0.78 | 0.78 | 0.78 |
| Q3 | 0.78 | 0.78 | 0.78 | 0.78 | 0.78 |
| **Sialylated carbohydrate antigen KL-‍6 (U/mL)** |  |  |  |  |  |
| n | 10 | 10 | 10 | 10 | 9 |
| Mean | 625.5 | 607.9 | 585.0 | 581.6 | 650.9 |
| SD | 656.3 | 600.1 | 522.1 | 511.5 | 683.6 |
| Q1 | 184.5 | 228 | 261.25 | 246 | 200 |
| Median | 352.5 | 349.5 | 340.5 | 350.5 | 392 |
| Q3 | 692.25 | 657.25 | 674.25 | 672.25 | 504 |
| **Matrix metalloproteinase-2 (ug/L)** |  |  |  |  |  |
| n | 9 | 9 | 9 | 9 | 8 |
| Mean | 151.00 | 155.67 | 164.36 | 166.22 | 198.85 |
| SD | 27.41 | 43.94 | 51.34 | 63.83 | 118.08 |
| Q1 | 137 | 116 | 120 | 123 | 120.5 |
| Median | 147 | 157 | 158 | 156 | 150 |
| Q3 | 157 | 198 | 193 | 170 | 251.75 |
| **Programmed cell death-1 (ng/L)** |  |  |  |  |  |
| n | 9 | 9 | 9 | 9 | 8 |
| Mean | 54.572 | 54.208 | 56.268 | 60.090 | 74.548 |
| SD | 23.425 | 24.896 | 25.259 | 36.389 | 52.339 |
| Q1 | 40.92 | 38.25 | 43.51 | 43.68 | 37.0425 |
| Median | 46.72 | 53.06 | 56.55 | 46.69 | 67.515 |
| Q3 | 60.7 | 64.79 | 64.55 | 70.51 | 86.66 |
| **RAGE (ng/L)** |  |  |  |  |  |
| n | 9 | 9 | 9 | 9 | 8 |
| Mean | 7096.90 | 3890.03 | 1959.80 | 1261.44 | 1159.04 |
| SD | 14074.66 | 6792.47 | 2230.86 | 870.53 | 600.98 |
| Q1 | 1130.5 | 762.8 | 732.3 | 703.4 | 705.175 |
| Median | 1620.9 | 1603.6 | 1004.4 | 964 | 1145.1 |
| Q3 | 4164.2 | 1774.7 | 1794.9 | 1498.8 | 1457.4 |
| **Chemokine ligand 5 (ng/L)** |  |  |  |  |  |
| n | 9 | 9 | 9 | 9 | 8 |
| Mean | 31081.1 | 31514.4 | 35870.0 | 32853.3 | 34275.0 |
| SD | 15396.6 | 14685.9 | 23980.8 | 27452.2 | 33464.4 |
| Q1 | 19700 | 26800 | 19100 | 18600 | 13975 |
| Median | 34400 | 31700 | 28800 | 21500 | 19400 |
| Q3 | 41500 | 36700 | 58000 | 35700 | 37725 |
| **SP-D (ug/L)** |  |  |  |  |  |
| n | 10 | 10 | 10 | 10 | 9 |
| Mean | 489.84 | 372.98 | 244.73 | 241.27 | 305.00 |
| SD | 963.17 | 425.73 | 195.12 | 191.44 | 330.47 |
| Q1 | 127.25 | 133.25 | 75.125 | 63.8 | 130 |
| Median | 151.5 | 180.5 | 197 | 205.5 | 168 |
| Q3 | 197 | 400.5 | 427.25 | 400.75 | 204 |
| **Transforming growth factor-β (ug/L)** |  |  |  |  |  |
| n | 10 | 10 | 10 | 10 | 9 |
| Mean | 4.036 | 3.615 | 2.919 | 2.606 | 3.833 |
| SD | 4.336 | 2.774 | 1.462 | 0.838 | 3.730 |
| Q1 | 2.03 | 2.0825 | 2.0675 | 2.2375 | 2.46 |
| Median | 2.205 | 2.68 | 2.35 | 2.43 | 2.85 |
| Q3 | 4.175 | 3.69 | 2.88 | 2.905 | 2.98 |
| **Thrombospondin-1 (ug/L)** |  |  |  |  |  |
| n | 9 | 9 | 9 | 9 | 8 |
| Mean | 15842.2 | 14981.1 | 13547.0 | 13805.6 | 11593.8 |
| SD | 8687.8 | 8369.0 | 7682.3 | 9648.5 | 7035.8 |
| Q1 | 12000 | 10200 | 9640 | 7050 | 6517.5 |
| Median | 13700 | 13700 | 10000 | 10200 | 8250 |
| Q3 | 21900 | 18700 | 20200 | 19900 | 19175 |

N denotes the number of participants in the analysis set; n denotes the number of participants with a measurement.

^a^Baseline is defined as the last measurement on day 0 pre-treatment or before (day –3 to –‍1).

*Q* quarter, *RAGE* receptor for advanced glycation end products, *SP-D* Surfactant Protein-D.
