# Supplementary material for: Clinical efficacy and safety of multipotent adult progenitor cells (invimestrocel) for acute respiratory distress syndrome (ARDS) caused by pneumonia: a randomized, open-label, standard therapy–controlled, phase 2 multicenter study (ONE-BRIDGE)
Source: Stem Cell Res Ther. 2023 Aug 22;14:217. doi: 10.1186/s13287-023-03451-z (PMC10464414; doi:10.1186/s13287-023-03451-z)
Supplement: Supplementary file 2 — Additional file 2. Methods. [file 13287_2023_3451_MOESM2_ESM.docx]

Additional file 2

Study population

*Inclusion criteria*

1. Provision of informed consent by the patient or his/her legal representative in case the patient is incapable of giving consent due to sedation, etc
2. Male or female aged 20–90 years at the time of informed consent (Asians only)
3. Patients with ARDS caused by pneumonia of those who were diagnosed as having ARDS according to the Berlin Definition
4. Patients who are confirmed to have the following findings in the Berlin Definition within the same 24 hours:

- Partial pressure arterial oxygen/fraction of inspired oxygen (PaO_2_/ F_I_O_2_) (P/F) ratio ≤300 mmHg with positive end-expiratory pressure (PEEP) ≥5 cm H_2_O
- Bilateral opacities on chest X-ray or computed tomography (not fully explained by effusions, lobar/lung collapse, or nodular shadow)
- Respiratory failure not fully explained by cardiac failure or fluid overload

1. Patients who underwent chest high-resolution computed tomography (HRCT)
2. Patients with HRCT score ≥211 according to the abbreviated HRCT scoring system
3. Patients with APACHE II score <27
4. Patients who were intubated and mechanically ventilated
5. Patients who can receive the investigational product within 72 hours (3 days) after the diagnosis of ARDS
6. Patients who are expected to have stable conditions during and immediately after administration. Stable condition is defined as a condition where there is no significant gradual increase in F_I_O_2_ or PEEP or that does not require cardiovascular support (e.g., an increase in the dose of norepinephrine or epinephrine by ≥0.1 μg/kg/min or an increase in the dose of vasoconstrictor or vasopressor other than norepinephrine and epinephrine by ≥20% to control blood pressure, etc)
7. Female patients who are not pregnant, breastfeeding, planning to become pregnant, or of childbearing potential. Female patients of childbearing potential who agree to use appropriate contraceptive methods under the guidance of the investigator (subinvestigator) until the end of the study
8. Male patients who have female partners of childbearing potential who agree to use appropriate contraceptive methods under the guidance of the investigator (subinvestigator) until the end of the study

*Exclusion criteria*

1. Patients who are not expected to survive for 48 hours
2. Patients on maintenance dialysis at screening
3. Patients with a life expectancy of <6 months due to complications at screening
4. Patients on mechanical ventilation at home due to chronic respiratory disease
5. Patients who have been on mechanical ventilation for ≥1 week
6. Patients with suspected acute exacerbation of chronic pulmonary fibrosis at screening
7. Patients with diffuse alveolar hemorrhage
8. Patients with chronic respiratory disease (interstitial lung disease or chronic obstructive pulmonary disease [COPD]) requiring continuous home oxygen therapy
9. Patients with severe COPD (GOLD stage III or higher)
10. Patients with chronic pulmonary hypertension (class III or IV according to the WHO classification of functional status of patients with pulmonary hypertension)
11. Patients with a history of lobectomy, single-lung pneumonectomy, or lung transplantation
12. Patients indicated for extracorporeal membrane oxygenation (ECMO) at screening
13. Patients resuscitated after cardiopulmonary arrest
14. Patients with a history of ST-elevation myocardial infarction within 6 months before informed consent
15. Patients with mean arterial pressure of <60 mmHg despite the use of ≥2 vasopressors regardless of the use or non-use of cardiotonic agent
16. Patients with severe chronic liver disease (Child-Pugh score >10)
17. Patients with a history of transplantation of autologous cells or allogeneic bone marrow/peripheral blood stem cells for purposes other than the treatment of hematological tumor
18. Patients with malignancy requiring treatment at screening
19. Patients infected with human immunodeficiency virus (HIV)
20. Patients with a history of acute allergic reaction to a human tissue/bovine/porcine-derived preparation, or those who refuse to use biologics for religious reasons
21. Patients for whom ARDS is not judged as the chief complaint by the investigator (sub-investigator) based on clinical findings
22. Patients who have used other investigational drugs or products within 30 days before informed consent
23. Patients who are participating or plan to participate in other clinical studies during the study period (except observational clinical studies that do not require intervention)
24. Patients who are judged by the investigator (sub-investigator) to be inappropriate for participation in this study due to significant complications (such as pneumothorax) or psychiatric diseases
25. Patients with highly suspected severe acute respiratory syndrome coronavirus 2 infection at screening

Standard treatment

Only pulmonary protective ventilation in respiratory management as listed in the table below could be performed during the study. Other therapies described in the ARDS clinical practice guidelines (Hashimoto S, et al: *J Intensive Care* 2017; 5:50) could be performed to the extent necessary, except for short-term high-dose methylprednisolone therapy and methylprednisolone pulse therapy.

| Treatment name | | Required | As necessary | Not allowed | Note |
| --- | --- | --- | --- | --- | --- |
| Respiratory management | Lung protective ventilation (targeting low tidal volume ventilation: 6–8 mL/kg [predicted body weight] and plateau pressure: ≤ 30 cm H_2_O) | X |  |  | Check data twice daily and adjust the ventilator to 6–8 mL/kg with the plateau pressure of 30 cm H_2_O |
|  | Use of muscle relaxants |  | X |  |  |
|  | Use of mild sedatives |  | X |  |  |
|  | Prone position therapy |  | X |  |  |
|  | Early rehabilitation |  | X |  |  |
|  | High-frequency oscillation (HFO) |  | X |  |  |
| Medication | Low-dose glucocorticoid therapy |  | X |  | Methylprednisolone  1 mg/kg/day |
|  | Short-term high-dose methylprednisolone therapy |  |  | X | Methylprednisolone  30 mg/kg QID for 1–2 days |
|  | Methylprednisolone pulse therapy |  |  | X | Methylprednisolone  1000 mg/day for 3 days |
|  | Neutrophil elastase inhibitors |  | X |  |  |
|  | Anticoagulation therapy  Antithrombin  Recombinant thrombomodulin  Heparin |  | X |  |  |
|  | Protease inhibitors  Nafamostat mesylate  Gabexate mesylate |  | X |  |  |
|  | NO inhalation therapy |  | X |  |  |
|  | Surfactants |  | X |  |  |
|  | Antioxidants |  | X |  |  |
|  | β-stimulants |  | X |  |  |
|  | Granulocyte-macrophage colony-stimulating factor (GM-CSF) |  | X |  |  |
|  | N-acetylcysteine |  | X |  |  |
|  | Statins |  | X |  |  |
|  | Antimicrobial/antifungal/antiviral drugs |  | X |  |  |
| Others | Extracorporeal membrane oxygenation (ECMO) |  | X |  | This can be performed if judged necessary due to changes in disease condition after enrollment |
|  | Endotoxin elimination therapy (PMX-F) |  | X |  |  |
|  | Renal replacement therapy |  | X |  | This can be performed if judged necessary due to changes in disease condition after enrollment |

Invimestrocel dose preparation and quality assurance

For this study, formulations of invimestrocel were available in vials that did not require processing in cell laboratories. Preparation of invimestrocel for infusion was conducted by trained staff competent with basic aseptic technique. Each vial of invimestrocel contained 3.2 mL (40 × 10^6^ cells/mL) of cell suspension. Eight vials were thawed in a 37°C water bath; thawing status was first checked after 5 minutes, and if invimestrocel was not thawed, the thawing status was checked every 30 seconds. Using a mini-spike dispensing pin (B. Braun Medical Inc., Bethlehem, PA, USA), as much liquid (3 mL) as possible was aspirated from seven vials, and approximately half (1.5 mL) was aspirated from the eighth vial. The aspirated product was injected into a bag containing 250 mL of a diluent having the same composition as Plasmalyte A (Baxter International Inc., Deerfield, IL, USA) to produce a final IV bag with the target cell dose for patient administration. The prepared product was stored at 2–8°C until patient administration and was administered at a rate of 10 mL/min using a Terumo TC-U300L infusion set (Terumo Corporation, Tokyo, Japan). The viability of the same lot of invimestrocel used in this study was 93.5–98.0% when re-thawed after freezing, and the long-term storage stability within the study period was also confirmed.

Ventilator weaning protocol

The below procedures were followed for confirmation and implementation of ventilator weaning:

1. Confirmation of the spontaneous breathing trial (SBT) safety screen.

SBT was performed if all the following criteria were met. If any criteria were not met, sedation was resumed and reassessment was conducted the next day.

- Improvement of causative disease
- No adverse events due to dose reduction/discontinuation of sedation
- Adequate oxygenation (SpO_2_ >90% under F_I_O_2_ ≤0.5 and PEEP ≤8 cm H_2_O)
- Stable circulatory dynamics (use of vasopressors allowed in small amounts)
- Ability to inhale

1. Implementation of SBT.

The following conditions were checked once daily. If the patient did not tolerate the following conditions, the conditions were set back to the pre-SBT state, the cause of inappropriateness was investigated, and adequate measures were taken.

- F_I_O_2_ ≤0.5
- Continuous Positive Airway Pressure (CPAP) ≤5 cm H_2_O (and pressure support [PS] ≤5 cm H_2_O) or T-piece
- Observation within 30 minutes to 2 hours

1. Success of SBT.

If all criteria were met, SBT was considered successful and extubation was considered or performed. If any of the criteria were not met, mechanical ventilation and sedation were resumed and the cause of the unsuccessful SBT was investigated. If the cause was identified, reassessment was conducted.

If SBT was successful but extubation was not performed, the reason was recorded.

- Respiratory rate <30 breaths/min
- No apparent decrease in respiratory parameters from baseline (e.g., SpO_2_ ≥94%, PaO_2_ ≥70 mmHg)
- Heart rate <140 bpm, with no evidence of new arrhythmia or myocardial ischemia
- No excessive increase in blood pressure
- No signs of respiratory distress (compared with pre-SBT status) given as follows:
- Excessive use of accessory muscle of respiration
- See-saw breathing (paradoxical breathing)
- Cold sweat
- Severe sensation of dyspnea, anxiety, or restlessness
